# A novel interstitial deletion of chromosome 2q21.1‐q23.3: Case report and literature review

**DOI:** 10.1002/mgg3.1135

**Published:** 2020-01-28

**Authors:** Bader Almuzzaini, Nasser S. Alatwi, Saif Alsaif, Mohammed A. Al Balwi

**Affiliations:** ^1^ Department of Medical Genomics Research King Abdullah International Medical Research Center Ministry of National Guard Health Affairs Riyadh Saudi Arabia; ^2^ Department of Pathology and Laboratory Medicine King Abdulaziz Medical City Ministry of National Guard Health Affairs Riyadh Saudi Arabia; ^3^ College of Medicine King Saud bin Abdulaziz University for Health Sciences Riyadh Saudi Arabia; ^4^ Department of Neonatal Intensive Care Unit King Abdulaziz Medical City Ministry of National Guard Health Affairs Riyadh Saudi Arabia

**Keywords:** 2q21.2‐q23.3, array Comparative Genomic Hybridization (aCGH), deletion KIF5C, Interstitial microdeletion, MBD5, ZEB2

## Abstract

**Background:**

Interstitial deletions of 2q are rare. Those that have been reported show varying clinical manifestations according to the size of the deletion and the genomic region involved.

**Method and Results:**

We describe a preterm male harboring a novel interstitial deletion encompassing the 2q21.2‐q23.3 region of 2q, a deletion that has not been described previously. The patient had multiple congenital anomalies including agenesis of the corpus callosum, congenital cardiac defects, bilateral hydronephrosis, spontaneous intestinal perforation, hypospadias and cryptorchidism, sacral dimple and rocker‐bottom feet. Array comparative genomic hybridization (aCGH) analysis revealed a de novo >18 Mb deletion at 2q21.1–q23.3, a region that included (605802, 611472 and 604593) OMIM genes.

**Conclusion:**

To the best of our knowledge this is the first report of a de novo interstitial deletion at 2q21.1–q23.3 in which haploinsufficiency of dose‐sensitive genes is shown to contribute to the patient's phenotype.

## Graphical abstract

Patient harboring a novel interstitial deletion encompassing 2q21.2‐q23.3 chromosomal region. Array revealed a de nova >18‐Mb deletion at 2q21.2‐q23.3. We thought that a haploinsufficiency genes may associated significantly contributed to patient manifestation.

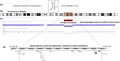

## 1 INTRODUCTION

There are more than 100 reports in the literature of terminal or interstitial deletions of 2q. The clinical manifestations vary according to the size of deletion and its location. Two relatively common small interstitial deletions have been reported: (a) deletion 2q21.1, characterized by mild intellectual disability, behavioral problems and a neurological disorder; and (a) deletion 2q22.1‐q22.3, characterized by sever intellectual disability (Bravo‐Oro et al., 2015; Gimelli et al., 2014; Greally, Robinson, Allen, O'Donovan, & Crolla, 2014; Mulatinho et al., 2012). A deletion involving 2q23.1 has been reported and this patient had short stature, learning and behavioral difficulty, autism and hyperactivity disorder (Mullegama et al., 2014; Noh & Graham, 2012; van Bon et al., 2010). Cases with interstitial deletion 2q31‐q33 showed distinctive dysmorphic features, developmental delay and severe mental retardation. Compared with high‐resolution chromosome banding by conventional karyotyping methodology, the exact breakpoints of deletions and duplications are delineated by Comparative Genomic Hybridization (aCGH). This has lead to the discovery of numerous microdeletion and microduplication syndromes (Ezugha et al., 2010; Lockwood, Chari, Chi, & Lam, 2006; Van Buggenhout et al., 2005).

In this presentation we describe a novel interstitial deletion encompassing 2q21.1‐q23.3 which was diagnosed by aCGH. The deleted region includes 56 genes, 28 of which are reported in OMIM database with particular impact of 12 genes on broadening the phenotypic spectrum. Among those genes there were dose‐sensitive genes that could manifest a phenotype when caused by haploinsufficiency, that is, *ZEB2* (OMIM 605802), *MBD5* (OMIM 611472), and *KIF5C* (OMIM 604593). Their individual contribution to the phenotype of the patient is discussed.

## 2 CLINICAL PRESENTATION

This male infant is the second child born to a healthy nonconsanguineous 29‐year‐old mother and a 31‐year‐old father. The proband has a 9‐year‐old healthy sister. Family history is unremarkable. Pregnancy care showed no history of exposure to teratogenic or infective agents. At end of the 35th week of gestation, the mother presented with premature rupture of membranes. An antenatal ultrasound scan detected multiple anomalies with normal 46, XY chromosomes on cordocentesis at low chromosomal banding resolution of 400.The preterm infant was born by spontaneous vaginal delivery at 36 weeks of gestation with a birth weight of 3.3 kg(50th centile), a length of 54cm (>95th centile) and head circumference of 33 cm(5‐10th centile). At birth examination, he had dysmorphic features that included hypertelorism, prominent nasal bridge, exophthalmos, low‐set ears with posterior rotation, micrognathia, short neck with redundant nuchal skin, clenched hands, hypospadias and cryptorchidism, sacral dimple, and rocker‐bottom feet (Table S1).

Shortly after birth the patient was transferred to the Neonatal Intensive Care Unit (NICU). Respiratory distress, spiked fever, and a cardiac murmur were detected. Echocardiogram (ECG) showed complex heart malformations consisting of double outlet right ventricle (DORV), ventricular septal defect (VSD), hypoplastic left ventricle and mitral valve, and Patent ductus arteriosus (PDA). Brain magnetic resonance imaging suggested total agenesis of the corpus callosum (Table S1).

His abdominal ultrasound showed abnormal configurations of the gallbladder with multiple septations and multiple intraluminal echogenic foci, and bilateral hydronephrosis (grade III on the right and grade II on the left). On the 2nd day of life the patient developed seizures, with episodes of status epilepticus, that responded to anticonvulsants medication.

After the first week of life the patient developed spontaneous intestinal perforation. He also developed clinical sepsis that required full sepsis work up and was treated with antibiotics. His hypoglycemia did not resolve with treatment. On the 2nd week of life the patient underwent a Laparotomy for multiple colon biopsies, ileostomy, appendectomy, and Meckel's diverticulum resection (Table S1). Investigations included skeletal survey, urinary amino and organic acids. All results were normal. In the 3rd week of life biopsy reports showed the presence of ganglion cells in the appendix, descending colon, transverse colon and splenic flexure. Persistent seizures required a high dose of anticonvulsant medication. He developed thrombosis of the right internal jugular vein and common iliac vein that required heparin administration. The patient also developed thrombocytopenia that required platelet transfusion. Complex congenital heart problems led to lung congestion that required prolonged high ventilation settings with inhaled nitric oxide therapy and full inotropic support. The patient died of hypoxic respiratory failure in the fourth week of life (Table S1).

## 3 METHODS

Detailed demographic and clinical data (symptoms, time of onset of symptoms, physical and radiological examination) and sample collections were obtained from the patient during his admission to NICU for medical evaluation and according to King Abdulaziz Medical City (KAMC) protocol following the Saudi Ministry of Health (MOH) guidelines. Samples for aCGH were obtained from the patient and his parents.

Both chromosomal studies and aCGH were performed at the division of Molecular Pathology and Genetics, Department of Pathology and Laboratory Medicine, KAMC, Riyadh.

High‐resolution chromosome (prometaphase) analysis on peripheral blood cultures was performed according to standard cytogenetic protocols following the International System for Human Cytogenetic Nomenclature (ISCN, 2016).

For array CGH analysis high‐quality genomic DNA was extracted from the collected peripheral blood using QIASymphonykit (Qiagen) following standard manufacturer's instructions. DNA quality was evaluated by NanodropND‐1000 (Thermo Wilmington), then fragmented, amplified, and hybridized using the CytoScan HD array platform (Affymetrix) and scanned with GeneChip scanner. Affymetrix CEL files were generated by the AffymetrixGeneChip® Command Console software (Affymetrix) and analyzed using the AffymetrixChASv3.2 software program. The software compares patient DNA hybridization to oligonucleotide and SNP probes of normal samples and the CNVs were mapped to the genome build GRCh38/hg19 for analysis and interpretation. In genomic imbalance, size of CNV, gene content and location were assessed in the interpretation. DECIPHER (https://decipher.sanger.ac.uk) (Firth et al., 2009), DGV (http://dgv.tcag.ca) (MacDonald, Ziman, Yuen, Feuk, & Scherer, 2014), ENSEMBL (https://asia.ensembl.org), OMIM (https://www.omim.org), and PubMed (https://www.ncbi.nlm.nih.gov/pubmed) were used to evaluate the significance of the detected CNVs.

## 4 RESULTS

**Figure 1:**
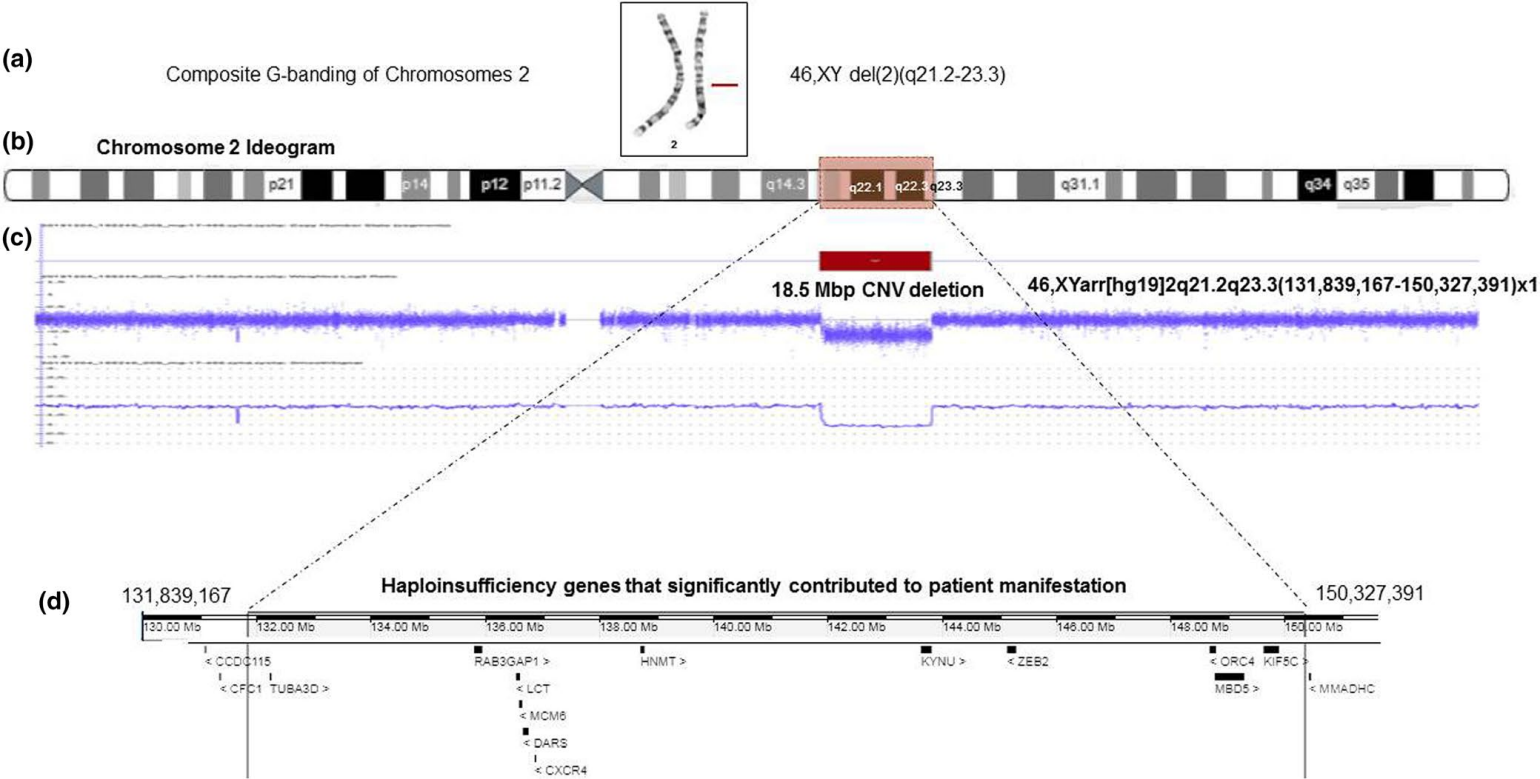
G‐banded chromosomes and array CGH analysis. High‐resolution chromosomal analysis revealed a male karyotype with 46,XY del(2)(q21.1q23.3) at banding resolution of 550. Chromosome 2 ideogram highlights the proposed microdeletion cytogenetic genomic segment. Array CGH illustrates the 18.5 Mbp CNV deletion that appears in red color. Haploinsufficiency genes that apparently contribute to the patient's phenotype

High‐resolution routine chromosomal analysis revealed a karyotype of 46,XY,del(2)(q23.1q23.3) at banding resolution of 550 (Figure 1a). A high‐resolution array CGH revealed a heterozygous interstitial deletion of 18,483 kb on cytogenetic band 2q21.1–23.3 (Figure 1b,c) at genomic position (chr2:131,839,167–150,327,391). The chromosomal constitution was reported as 46,XY.arr[GRCH38]2q21.2q23.3(131,839,167_150,327,391)×1. This 18.5 Mb deletion encompasses 56 genes. Array CGH on the parents was normal.

## 5 DISCUSSION

**Figure 2:**
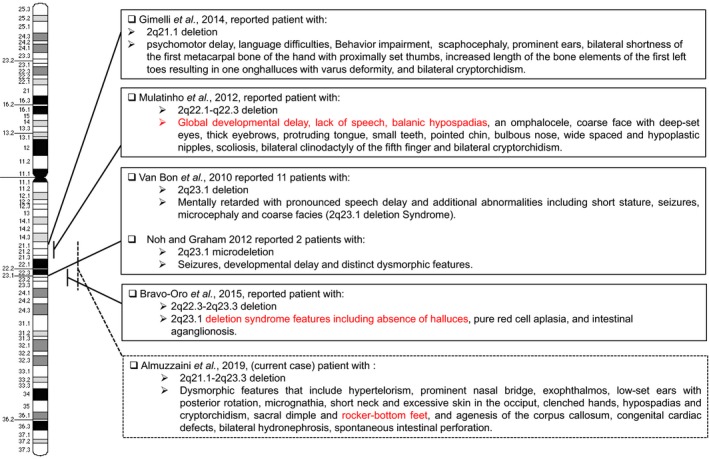
Summary of significant reported cytogenetic breakpoints associated with chromosome 2 band q21.1‐23.3

We believe that this novel interstitial deletion encompassing 2q21.1‐q23.3 genomic chromosomal regions has contributed significantly to the patient's phenotype. The cytogenetic breakpoints and phenotype of patients with reported deletions within region 2q21.1‐q23.3 are summarized in Figure 2. In our patient, array CGH analysis showed a deletion of approximately 18.5Mb at 2q21.1‐q23.3, a region that includes 56 genes with 38 protein‐coding genes. Of those genes, ZEB2, *MBD5,* and *KIF5C* cause recognizable clinical syndromes when one allele is mutated or deleted (Table S2).

Haploinsufficiency of *ZEB2* causes Mowat‒Wilson syndrome [MWS, OMIM 235730], a syndrome characterized by intellectual disability, delayed motor development, epilepsy, microcephaly, characteristic facial features, heart defects, genitourinary malformations, and Hirschsprung disease (Kluk et al., 2011; Mowat et al., 1998; Mowat, Wilson, & Goossens, 2003). Various sizes of deletions have been reported describing a MWS cases with deletion distal or upstream to the ZEB2 gene, proposing a possible disturbance of regulatory elements (Ballarati et al., 2009; Bravo‐Oro et al., 2015; Garavelli et al., 2009; Mowat et al., 2003; Saunders, Zhao & Ardinger, 2009).

MBD5 haploinsufficiency is associated with typical manifestations of 2q23.1 microdeletion syndrome [OMIM 611472] (Talkowski et al., 2011) which is characterized by variable clinical presentations including microcephaly, intellectual disability, seizures, mild dysmorphic features, behavioral issues, and autistic‐like features.

KIF5C heterozygous missense mutations are associated with complex cortical dysplasia and other brain malformations [OMIM 615282](Poirier et al., 2013; Willemsen et al., 2014).

## 6 CONCLUSION

We describe a de novo interstitial deletion of 2q21.2‐2q23.3. This genomic segment contains 56 morbid genes, 38 of which have a reported phonotype in OMIM. Our patient has some clinical findings described in patients with deletion of ZEB2, MBD5, and KIF5C genes but he also has clinical findings not previously described in patients with deletions involving this region of 2q. This may be due to some, as yet, undiscovered genes in the region or may be caused by other factors such as epigenetic effects occurring as a result of this large deletion.

## CONFLICT OF INTEREST

None declared.
