# Supplementary material for: A novel interstitial deletion of chromosome 2q21.1‐q23.3: Case report and literature review
Source: Mol Genet Genomic Med. 2020 Jan 28;8(4):e1135. doi: 10.1002/mgg3.1135 (PMC7196451; doi:10.1002/mgg3.1135)
Supplement: Supplementary file 1 [file MGG3-8-e1135-s001.docx]

**Supplementary Table 1.** Summary of patient’s detailed clinical manifestation

| **Central Nervous System** | Electroencephalogram (EEG) showed occasional independent sharp waves with very low amplitude background activity suggestive of a diffuse encephalopathy of none specific aetiology.  Neonatal Head US - Corpus callosum agenesis.  Brain MRI -   1. Total agenesis of the corpus callosum 2. Small subdural haemorrhage |
| --- | --- |
| **cardiovascular system** | Echocardiogram (ECHO)   - Double outlet right ventricle (DORV) with both great arteries arising from the right ventricle. - Aorta slightly anterior and to the right to the pulmonary artery. - Small outlet ventricular septal defect and large apical muscular   VSD   - Large tortuous patent ductus arteriosus (PDA) with left to right shunt. - Unobstructed left aortic arch, cannot rule out coarctation of the aorta in the presence of large PDA. - Good left ventricular systolic function. |
| **Respiratory** | Acceptable sat 80% and above - as per cardiology  ON HFVO MODE MAP=18,DELTA PRESSURE OF 38,HZ=8 Fio2 80 iNO 2  on daily CXR and CBG q12  chest x-ray: lung congestion was on furosemide and captopril as per cardiology advice (to decrease afterload) |
| **Gastrointestinal System** | spontaneous bowel perforation in first week of life, operated next day  - Exploratory laparotomy during which found perforations at the splenic flexure of the colon and distal sigmoid, which was macerated. coincidental finding of Meckel diverticulum, Multiple colonic biopsies was taken, Appendectomy done, Meckel’s Diverticulum incision and Ileostomy was seen daily by Paediatric Surgery |
| **Genitourinary System** | pass urine, Bilateral hydronephrosis Grade III Rt side and grade II Lt side  catheter was inserted by urologist in OR after urethral dilation |
| **Hematology** | - Right common iliac vein, external iliac vein, and common femoral thrombosis (on heparin protocol) - thrombocytopenia on frequent transfusion |
| **Infectious diseases** | Spikes of fever underwent full septsis work up early in life completed 14 day Meropenem and 7 days vancomycin, currently on another course of abx Vancomycin and Meropenem 10 days total, No positive cultures was ever reported |
