# Supplementary material for: A novel interstitial deletion of chromosome 2q21.1‐q23.3: Case report and literature review
Source: Mol Genet Genomic Med. 2020 Jan 28;8(4):e1135. doi: 10.1002/mgg3.1135 (PMC7196451; doi:10.1002/mgg3.1135)
Supplement: Supplementary file 2 [file MGG3-8-e1135-s002.docx]

**Supplement Table 2.** Genes described in OMIM and associated with 2q21.1-q23.3 cytoband genomic segment

| **Gene (OMIM)** | **Related phenotype** |
| --- | --- |
| GPR39 (602886) | Unknown |
| LYPD1 (610450) | Unknown |
| NCKAP5 (608789) | Unknown |
| MGAT5 (601774) | Unknown |
| ACMSD (608889) | Unknown |
| CCNT2 (603862) | Unknown |
| RAB3GAP1 (602536) | Warburg micro syndrome (AR) |
| ZRANB3 (615655) | Unknown |
| MIR128-1 (611774) | Unknown |
| UBXN4 (611216) | Unknown |
| LCT (603202) | Lactase deficiency, congenital (AR) |
| MCM6 (601806) | Lactase persistence/nonpersistence (AD) |
| DARS (603084) | Hypomyelination with brainstem and spinal cord involvement and leg spasticity (AR) |
| CXCR4 (162643) | WHIM syndrome with neutropenia |
| HNMT (605238) | Mental retardation (AR)  Mental retardation (AR) |
| NXPH2 (604635) | Unknown |
| LRP1B (608766) | Unknown |
| KYNU (605197) | Hydroxykynureninuria (AR),  Vertebral, cardiac, renal, and limb defects syndrome (AR) |
| ARHGAP15 (610578) | Unknown |
| GTDC1 (610165) | Unknown |
| ZEB2 (605802) | Mowat-Wilson syndrome (AD) ,mental retardation, hypertelorism, submucous cleft palate, and short stature |
| ACVR2A (102581) | Unknown |
| ORC4 (603056) | Meier-Gorlin syndrome (AR) |
| MBD5 (611472) | Mental retardation (AD) |
| EPC2 (611000) | Unknown |
| KIF5C (604593) | Cortical dysplasia, complex, with other brain malformations. Three cases reported as IUD,One surviving son who presented with microcephaly arthrogryposis, and early-onset epilepsy, and he was bedridden with spastic tetraplegia at age 1 month. Brain MRI showed frontal and perisylvian polymicrogyria and thin corpus callosum. The findings extended the association between microtubule-based cellular processes and proper cortical development  [Poirier et al. (2013)](https://www.omim.org/entry/604593?search=KIF5C%28604593%29&highlight=kif5c%20604593#5) |
| LYPD6 (613359) | Unknown |
| MMADHC (611935 | Homocystinuria (AR)  Methylmalonic aciduria and homocystinuria (AR)  Methylmalonic aciduria (AR) |
